# The phytase RipBL1 enables the assignment of a specific inositol phosphate isomer as a structural component of human kidney stones†

**DOI:** 10.1039/d2cb00235c

**Published:** 2023-01-27

**Authors:** Guizhen Liu, Esther Riemer, Robin Schneider, Daniela Cabuzu, Olivier Bonny, Carsten A. Wagner, Danye Qiu, Adolfo Saiardi, Annett Strauss, Thomas Lahaye, Gabriel Schaaf, Thomas Knoll, Jan P. Jessen, Henning J. Jessen

**Affiliations:** a Institute of Organic Chemistry & CIBSS-Centre for Integrative Biological Signalling Studies, University of Freiburg Germany henning.jessen@oc.uni-freiburg.de; b Institute of Crop Science and Resource Conservation, Department of Plant Nutrition, University of Bonn Germany; c Department of Biomedical Sciences, University of Lausanne, Switzerland and Service of Nephrology, Lausanne University Hospital Lausanne Switzerland; d Institute of Physiology, University of Zürich Switzerland; e Laboratory for Molecular Cell Biology, University College London UK; f ZMBP, University of Tübingen Germany; g Department of Urology, Sindelfingen-Boeblingen Medical Center, Teaching Hospital University Tübingen Sindelfingen Germany

## Abstract

Inositol phosphates (InsPs) are ubiquitous in all eukaryotes. However, since there are 63 possible different phosphate ester isomers, the analysis of InsPs is challenging. In particular, InsP_1_, InsP_2,_ and InsP_3_ already amass 41 different isomers, of which some occur as enantiomers. Profiling of these “lower” inositol phosphates in mammalian tissues requires powerful analytical methods and reference compounds. Here, we report an analysis of InsP_2_ and InsP_3_ with capillary electrophoresis coupled to electrospray ionization mass spectrometry (CE-ESI-MS). Using this method, the bacterial effector RipBL1 was analyzed and found to degrade InsP_6_ to Ins(1,2,3)P_3_, an understudied InsP_3_ isomer. This new reference molecule then aided us in the assignment of the isomeric identity of an InsP_3_ while profiling human samples: in urine and kidney stones, we describe for the first time the presence of defined and abundant InsP_3_ isomers, namely Ins(1,2,3)P_3_, Ins(1,2,6)P_3_ and/or Ins(2,3,4)P_3_.

## Abstract 2

Kidney stones and patient urine contain inositol 1,2,3-trisphosphate as demonstrated by capillary electrophoresis mass spectrometry with an internal heavy isotope reference.

## Graphical abstract

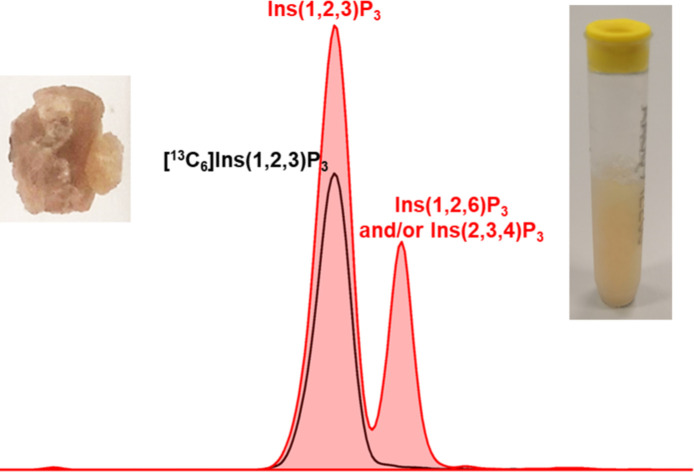

## Introduction

*myo*-Inositol phosphates (InsPs) are molecules with various numbers of phosphate groups modified on one to six of the OH groups of *myo*-inositol (hereafter Ins), which are present in all eukaryotes. By sequential phosphorylation, 63 different InsPs can in principle be generated, of which over one third are thought to be relevant to mammalian metabolism as signalling molecules.^1,2^

Analysis of these InsPs is important to better characterize InsP identity and abundance in mammalian metabolism, which might finally lead to a deciphering of the alleged “inositol phosphate code”.^1,3,4^ A variety of analytical methods have been reported, including, for example, strong anion exchange high performance liquid chromatography (SAX-HPLC), high performance liquid chromatography coupled to mass spectrometry (HPLC-MS), and capillary electrophoresis coupled to electrospray ionization mass spectrometry (CE-ESI-MS).^5–13^ Most of these methods are sensitive and efficient for the analysis of highly phosphorylated InsPs (*e.g.* InsP_4_, InsP_5_, InsP_6_).^5–11^ Moreover, CE-MS methods are powerful tools for profiling of inositol pyrophosphates (PP-InsPs), such as InsP_7_ and InsP_8_ that carry one or several diphosphate groups.^6,14–16^

Analyzing identity and abundance of InsP_1_, InsP_2_ and InsP_3_ isomers in biological samples remains a significant challenge for several reasons. First, due to the non-specificity of the current extraction and separation methods, it is difficult to identify InsP_1_ and InsP_2_ isomers from the isobaric and more abundant sugar mono- and diphosphates. Second, the number of possible InsP_1_ to InsP_3_ isomers amasses to 41 and out of these alone 20 isomers of InsP_3_ exist, including 8 enantiomeric pairs. Enantiomer separation on a chiral stationary phase remains an unsolved issue for these molecules. Additionally, it is generally assumed that the *myo*-configuration is the relevant one, but also other inositol configurations occur in biology.^17^ Moreover, the high negative charge density and the absence of a chromophore generate significant issues regarding sensitive detection. The most commonly used analytical methods for InsP_2_ and InsP_3_ separation are SAX-HPLC and HPLC-MS.^9,18,19^ The latter can profit from a heavy isotope labelling methylation strategy to enable ESI^+^ measurements.^9^ Ins(1,4,5)P_3_, the Ca^2+^ release factor,^20^ is the most characterized InsP_3_ and represents the textbook example of a second messenger. Other InsP_3_ isomers have been described in biology, such as Ins(1,3,4)P_3_ and Ins(1,2,3)P_3_.^18^ InsP_(1–5)_ with a phosphate group in the 2-position are usually not described in mammalian metabolite overviews,^2,21–24^ although such esters of InsP_2_ and InsP_3_ have been found since 1995.^18,25,26^ Recently, using ^13^C enrichment and 2D-NMR analysis, it was discovered that Ins(2,3)P_2_ and Ins(2)P are major metabolites in immortalized mammalian cell lines, calling into question the general notion that InsPs with a phosphate in the axial 2-position are biologically irrelevant.^27^ A recent finding that 2-PP-InsP_5_ carrying a pyrophosphate in the 2-position is a biologically relevant species, further underscores the need to reassess inositol phosphate structures.^16^ Recently developed new analytical technologies are now able to reveal several ‘discarded’ isomers, whose biological importance must now be evaluated.

Our approach to analyze InsP_4–6_ and PP-InsPs by CE-ESI-MS is extended herein to study InsP_2–3_. We were interested to profile human samples and since the involvement of PP-InsPs in systemic human phosphate homeostasis has become clearer,^28–31^ we decided to analyze urine samples where excess phosphate is excreted. Surprisingly, we detected several InsP_3_ isomers in urine. This led us to postulate that these InsP_3_ might be structural components of kidney stones. If this would be the case, we could potentially use urine InsP presence as a biomarker for impending stone formation. To achieve our objectives, we reveal that RipBL1, a bacterial phytase effector protein,^32^ selectively dephosphorylates InsP_6_ to Ins(1,2,3)P_3_. Our CE-ESI-MS method combined with [^13^C_6_] InsP_3_ produced from [^13^C_6_] InsP_6_^33,34^ by RipBL1 now enables us to assign InsP_3_ in human kidney stone and urine and demonstrates in patient samples (healthy *vs.* kidney stone formers) that the most abundant isomers host a phosphate in the 2-position.

## Results

### InsP_2_ and InsP_3_ isomers are well separated by CE-ESI-MS

The CE-ESI-MS analytical method has been originally developed to investigate inositol pyrophosphate metabolism and is a particularly effective separation platform for isomers of InsP_4–6_ and also for PP-InsPs.^6^ CE-ESI-MS was not further developed to analyze InsP_1–3_ in biological samples, despite the potential for the determination of spiked InsP_3_ in plasma by capillary zone electrophoresis-mass spectrometry.^12^ There are 15 isomers of InsP_2_ that include 6 enantiomeric pairs, and 20 InsP_3_ isomers that include 8 enantiomeric pairs. We disclose a CE-ESI-MS method for analyzing six commercially available isomers of InsP_2_ and seven commercially available isomers of InsP_3_ by baseline separation, other than enantiomers, which one cannot discriminate by using an achiral bare fused silica capillary.

**Fig. 1:**
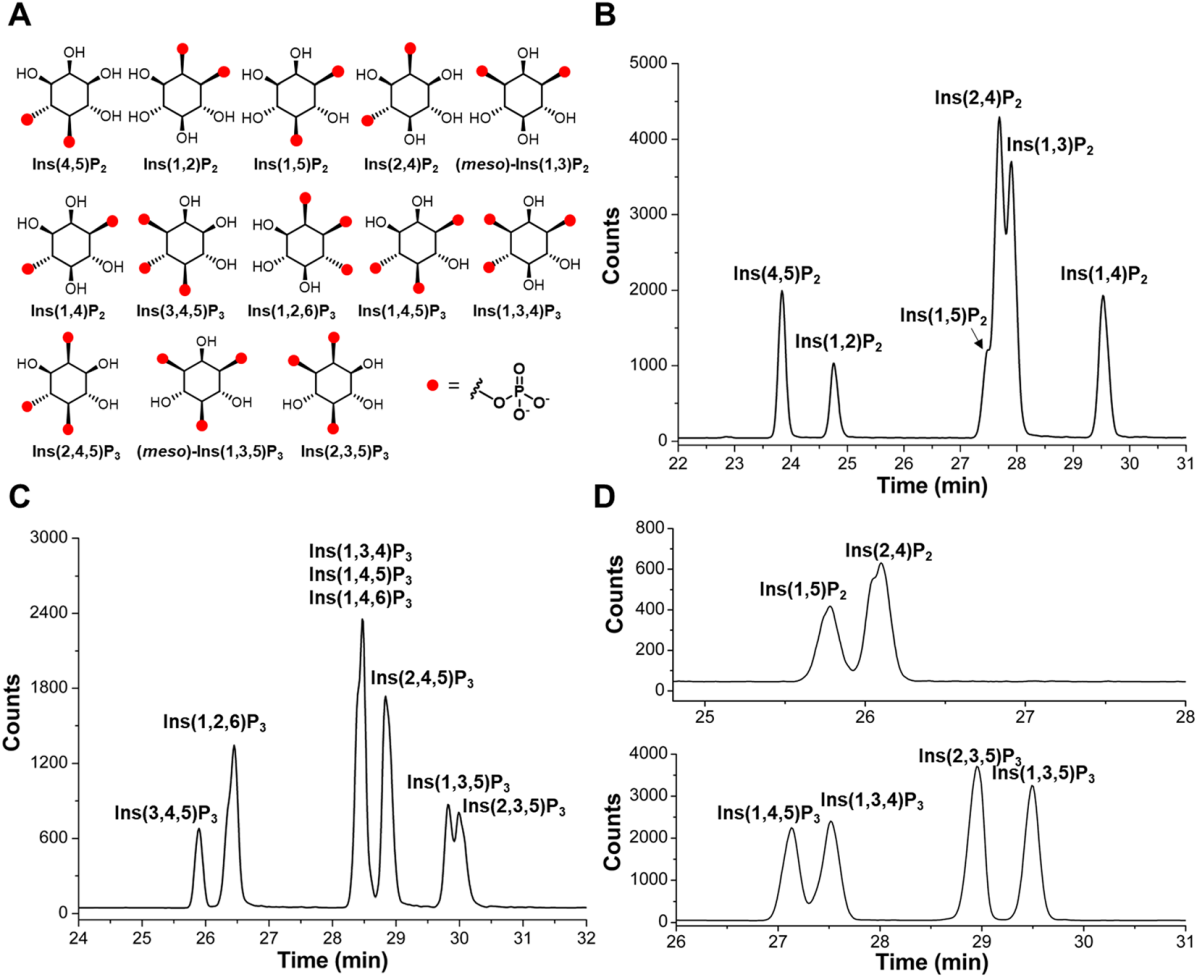
Separation of InsPs by CE-ESI-MS. (A) Structures of commercial InsP_2_ and InsP_3_ isomers. Achiral isomers are labeled as “*meso*”. (B and C) Separation of InsP_2_ and InsP_3_ standards by CE-ESI-MS, BGE: 35 mM ammonium acetate titrated with ammonium hydroxide to pH 9.75. (D) Separation of InsP_2_ and InsP_3_ standards by CE-ESI-MS, BGE: 50 mM ethylamine titrated with formic acid to pH 10.0.

The set of commercial InsP_2_ and InsP_3_ are shown in Fig. 1A: Ins(4,5)P_2_, Ins(1,2)P_2_, Ins(1,5)P_2_, Ins(2,4)P_2_, Ins(1,3)P_2_, Ins(1,4)P_2_, Ins(3,4,5)P_3_, Ins(1,2,6)P_3_, Ins(1,3,4)P_3_, Ins(1,4,5)P_3_, Ins(2,4,5)P_3_, Ins(1,3,5)P_3_, Ins(2,3,5)P_3_. A bare fused silica capillary with a length of 100 cm was implemented for separations by applying 30 kV across the capillary (Agilent 7100 CE). Detection was achieved with an ESI-QQQ-MS (Agilent 6495C Triple Quadrupole with Agilent Jet Stream electrospray ionization source) in the negative ionization mode connected with an Agilent CE-ESI-MS interface. Initially, 35 mM ammonium acetate titrated with ammonium hydroxide to pH 9.75 was used as background electrolyte (BGE). With this BGE, separation of six InsP_2_ isomers is achieved, but Ins(1,5)P_2_, Ins(2,4)P_2_, and Ins(1,3)P_2_ were not baseline separated (Fig. 1B). Also, the separation of eight InsP_3_ isomers was achieved with the exception of Ins(1,3,4)P_3_, Ins(1,4,5)P_3_, and Ins(1,4,6)P_3_ (Fig. 1C). After pH optimization and BGE screening, a near baseline separation of Ins(1,5)P_2_ and Ins(2,4)P_2_ was achieved with 50 mM ethylamine titrated with formic acid to pH 10.0 (Fig. 1D). Separation of previously coeluting Ins(1,3,4)P_3_ and InsP(1,4,5)P_3_ was also resolved using this BGE (Fig. 1D) and a baseline separation of InsP(1,3,5)P_3_ and Ins(2,3,5)P_3_ was achieved as well (Fig. 1D). Ins(1,4,6)P_3_ still coeluted with Ins(1,4,5)P_3_.

Method validation was performed with InsP standards, including linearity, limit of detection (LOD) and limit of quantification (LOQ). The external calibration curves of InsP_2_ and InsP_3_ were constructed at eight concentration levels by regression of concentrations against the analyte peak area (Fig. S1, ESI†). The calibration curves were linear and had a coefficient of determination >0.997 over the investigated range of 0.1–10 μg mL^−1^ for Ins(1,2)P_2_ and 0.4–40 μg mL^−1^ InsP(1,2,6)P_3_. With 20 nL sample injection, the LODs are 0.025 μg mL^−1^ for InsP_2_ (*i.e.* 1.3 fmol) and 0.020 μg mL^−1^ for InsP_3_ (*i.e.* 0.8 fmol), the LOQs are 0.05 μg mL^−1^ for InsP_2_ (*i.e.* 2.6 fmol) and 0.10 μg mL^−1^ for InsP_3_ (*i.e.* 4 fmol; Fig. S1, ESI†). The limit of detection of this method is significantly lower compared to other chromatography-related methods summarized in ref. 35.

### RipBL1 degrades InsP_6_ to Ins(1,2,3)P_3_

**Fig. 2:**
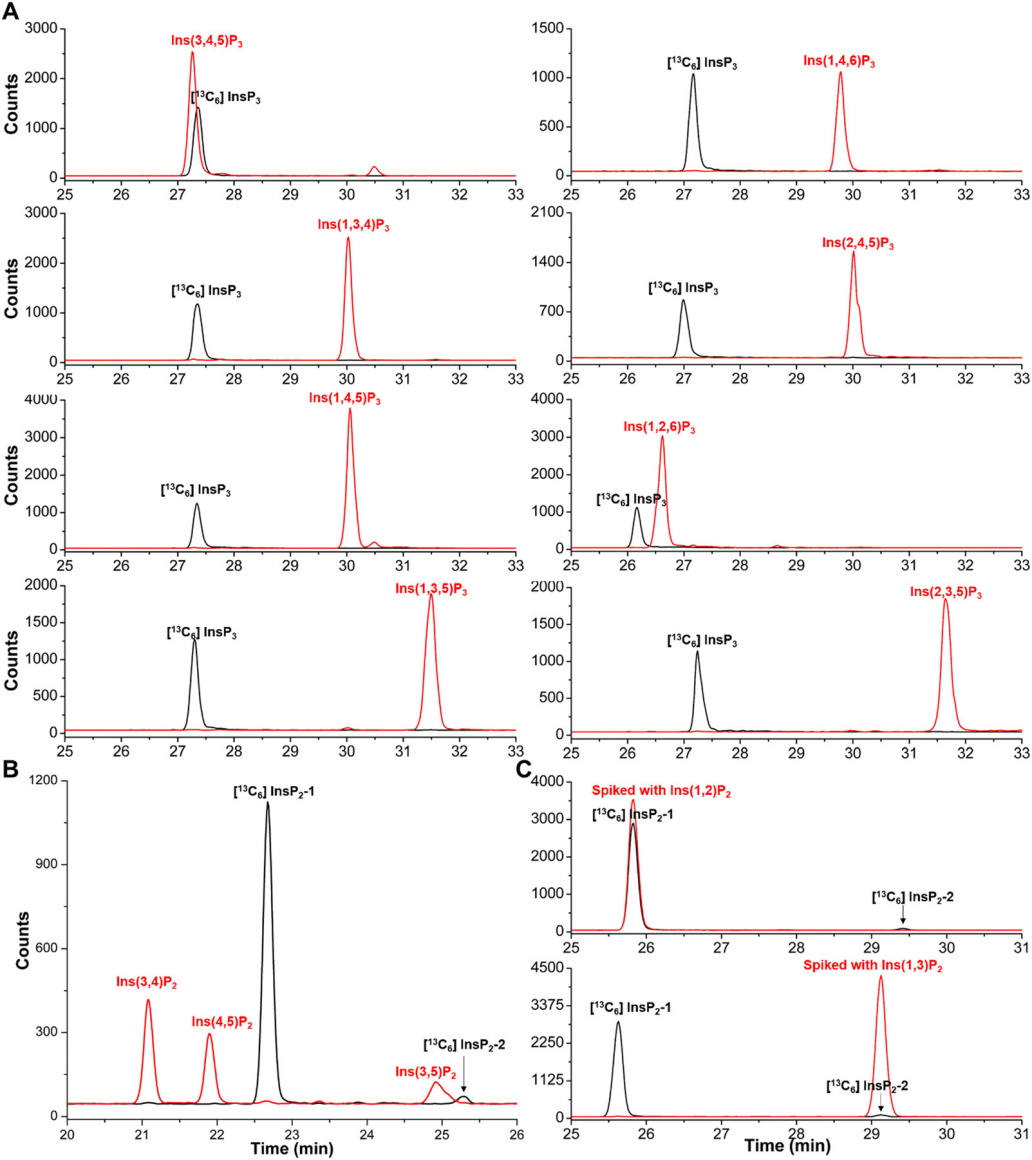
Identification of the InsP_3_ (black line) dephosphorylation product of [^13^C_6_] InsP_6_ by the RipBL1 enzyme. (A) CE-ESI-MS analysis of InsP_3_ individually spiked (red line) with Ins(3,4,5)P_3_ standard, Ins(1,4,6)P_3_ standard, Ins(1,3,4)P_3_ standard, Ins(2,4,5)P_3_ standard, Ins(1,4,5)P_3_ standard, Ins(1,2,6)P_3_ standard, Ins(1,3,5)P_3_ standard or Ins(2,3,5)P_3_ standard, as indicated. (B) Analysis of InsP_2_ generated from Ins(3,4,5)P_3_ and the [^13^C_6_] InsP_3_ isomer by heating to 100 °C for 2.5 h. Extracted ion electropherograms of [^13^C_6_]-labelled InsP_2_ (black lines) generated by [^13^C_6_] InsP_3_ and InsP_2_ (red trace) generated by Ins(3,4,5)P_3_. (C) [^13^C_6_] InsP_2_ (black line) generated from [^13^C_6_] InsP_3_ after heating to 100 °C for 2.5 h spiked with Ins(1,2)P_2_ standard or Ins(1,3)P_2_ standard as indicated (red line). Note that the [^13^C_6_] InsP_2_-1 isomer has the same migration time as the Ins(1,2)P_2_ standard and that the [^13^C_6_] InsP_2_-2 isomer has the same migration time as the Ins(1,3)P_2_ standard.

RipBL1 is an effector protein of the phytopathogenic Gram-negative bacteria *Ralstonia solanacearum*, that shares homology with the bacterial effector XopH from *Xanthomonas campestris*. Previous work demonstrated that XopH displays an unusual phytase activity that dephosphorylates InsP_6_ stereoselectively at the C1 position only, resulting in the generation of Ins(2,3,4,5,6)P_5_.^32^ We tested InsP_6_ dephosphorylation by phytases, such as RipBL1 *in vitro*, to see if we could generate more high-quality non-commercial standards. Importantly, after 45 min 100% of InsP_6_ was degraded by RipBL1, and one defined InsP_3_ was detected as constituting about 95% of the digestion product. There was very little dephosphorylation to InsP_4_ and InsP_2_ (Fig. S2B, ESI†). CE-qTOF-MS analyses of the enzymatic reaction product confirmed a main molecular ion peak at *m*/*z* 418.9551 corresponding to InsP_3_ (Fig. S2A, ESI†). To structurally assign the InsP_3_ product, we generated a [^13^C_6_] InsP_3_ by [^13^C_6_] InsP_6_^33^ digestion with RipBL1, and then analyzed its identity through spiking-in of all commercially available InsP_3_ standards. As shown in Fig. 2A, the [^13^C_6_] InsP_3_ was baseline separated from all commercially available InsP_3_ standards, with the exception of Ins(3,4,5)P_3_ but also for this isomer, there was no perfect comigration. Previous work suggests that pyrohydrolysis can partially degrade “higher” InsPs and generate”lower” InsPs through dephosphorylation, and the pyrohydrolysis does not cause phosphate migration.^19,36^ Analysis of pyrohydrolysis products of Ins(3,4,5)P_3_ and the [^13^C_6_] InsP_3_ solution after heating to 100 °C for 2.5 h confirmed that [^13^C_6_] InsP_3_ is different from Ins(3,4,5)P_3_, as the labeled *vs.* non-labeled InsP_2_ products were different (Fig. 2B).

As Ins(4,5,6)P_3_, Ins(2,3,6)P_3_ (or its enantiomer Ins(1,2,4)P_3_), as well as the *meso*-compounds Ins(1,2,3)P_3_, and Ins(2,4,6)P_3_ were not readily available, the further assignment was performed differently. We compared the migration time of [^13^C_6_] InsP_3_ with the pyrohydrolysis products of Ins(2,3,5,6)P_4_ and Ins(1,4,5,6)P_4_ solution, respectively. The [^13^C_6_] InsP_3_ product of RipBL1 does not comigrate with any InsP_3_ isomers found as pyrohydrolysis products of Ins(2,3,5,6)P_4_ or Ins(1,4,5,6)P_4_ (Fig. S3, ESI†). These results provide evidence that the identity of the [^13^C_6_] InsP_3_ product is either Ins(1,2,3)P_3_ or Ins(2,4,6)P_3_, both of which are symmetric *meso*-compounds.

Pyrohydrolysis products of Ins(1,2,3)P_3_ and Ins(2,4,6)P_3_ are expected to be different. For Ins(1,2,3)P_3_, three InsP_2_, namely Ins(1,2)P_2_, Ins(2,3)P_2_ and Ins(1,3)P_2_, should be the pyrohydrolysis products. On the other hand, Ins(2,4)P_2_, Ins(2,6)P_2_, and Ins(4,6)P_2_ would be the expected pyrohydrolysis products of Ins(2,4,6)P_3_. By comparing the electrophoretic mobility with the commercially available InsP_2_ standards, two InsP_2_ peaks were identified as Ins(1,2)P_2_ and its enantiomer Ins(2,3)P_2_ (major product) as well as Ins(1,3)P_2_ (minor product) in the pyrohydrolysis solution of [^13^C_6_] InsP_3_ (Fig. 2C). Consequently, [^13^C_6_] InsP_3_ generated by RipBL1 treatment of [^13^C_6_] InsP_6_ represents Ins(1,2,3)P_3_.

Dephosphorylation of InsP_6_ by different types of phytases to produce Ins(1,2,3)P_3_, either as the final product or as intermediates, has been investigated.^37–41^ Ins(1,2,3)P_3_ as an intermediate in barley aleurone tissue was described as well.^42^ That InsP_6_ metabolism by phytase in plants and fungi might pass through Ins(1,2,3)P_3_ was discussed.^18^ Ins(1,2,3)P_3_ as the final product of InsP_6_ hydrolysis, as shown for RipBL1, is however unusual, only alkaline phytase from Lily Pollen is a candidate enzyme but the Ins(1,2,3)P_3_ purity was not described.^38^ Thus, to the best of our knowledge, RipBL1 is the first bacterial effector phytase that with high selectivity and yield generates Ins(1,2,3)P_3_.

### Ins(1,2,3)P_3_, Ins(1,2,6)P_3_ and/or its enantiomer Ins(2,3,4)P_3_ exist in human kidney stones and urine

Kidney stones are solid objects in the kidney or bladder that can cause different disease symptoms and consist of various low molecular weight compounds as well as proteins.^43^ Further, kidney stones can be categorized into calcium containing stones and non-calcium containing stones. Calcium containing stones are the most common forms of kidney stones, including calcium oxalate monohydrate (COM) or dihydrate (COD) as well as calcium phosphate and mixtures of these.^43^ The formation of kidney stones is mainly driven by urinary supersaturation and crystallization. These processes are environment dependent, influenced by urine pH, concentration of specific substances and effective molecules (promoters, and inhibitors of kidney stone formation).^44^ Studies have shown that for example urinary InsP_6_ can inhibit crystallization during the process of kidney stone formation in a model system.^45–48^ Recently, InsP_6_ analogues with PEG modifications were reported to completely inhibit such crystallization processes in the nanomolar range.^49^ Additionally, studies with SNF472 (a hexasodium salt of InsP_6_) as an inhibitor of vascular calcification in a phase 2 clinical trial are ongoing,^50^ highlighting a strong relationship between InsP_6_ and calcification. Whether some other inositol phosphates might actually promote crystallization and whether these substances are then incorporated into the kidney stone has not been studied. We therefore asked the question if InsP_6_ or other InsPs could be structural components of kidney stones.

**Fig. 3:**
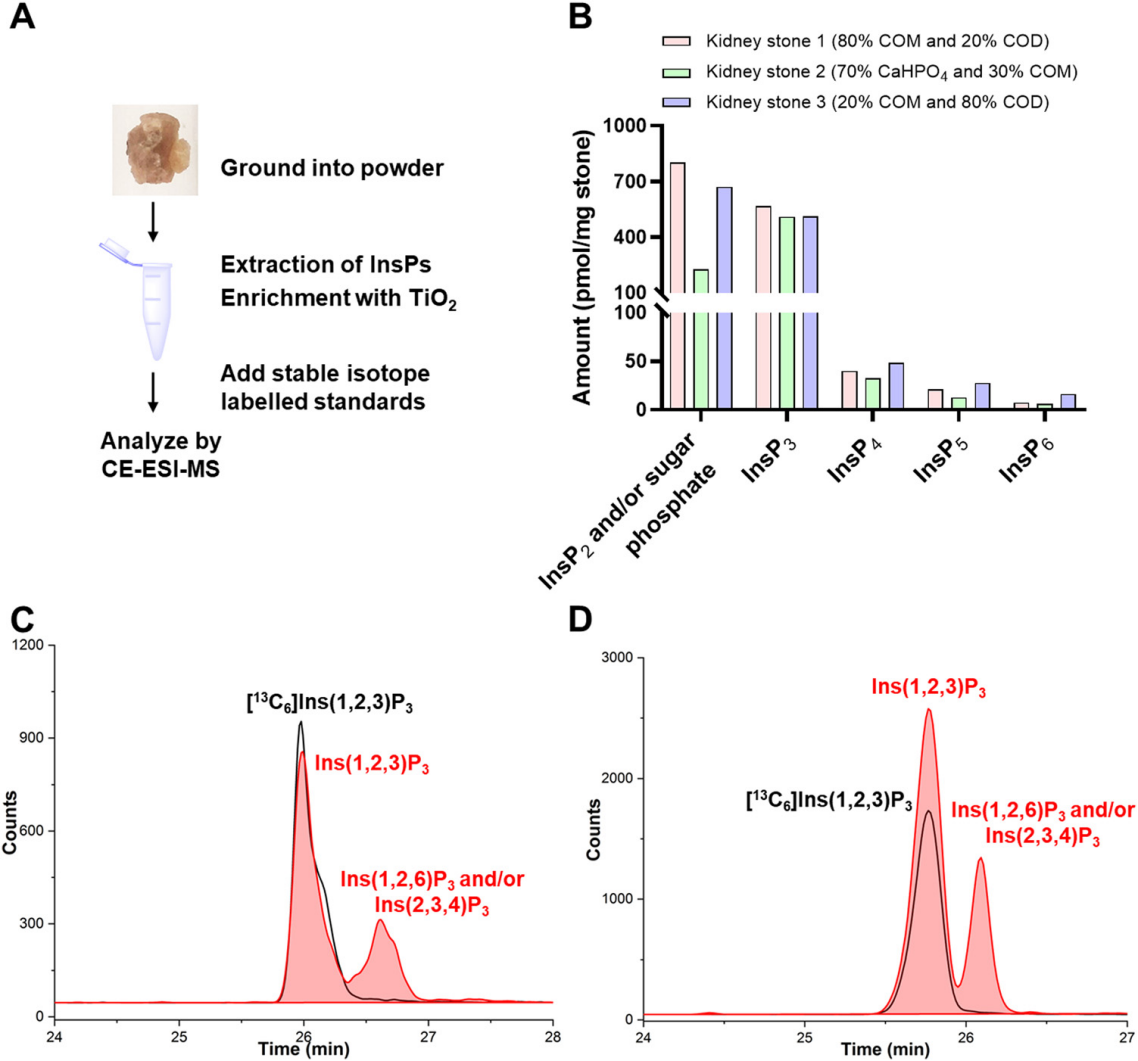
Profiling of InsP_s_ in human kidney stones. (A) Extraction and analysis workflow of kidney stones for CE-ESI-MS. (B) InsPs distribution in kidney stone 1 (contains 80% COM and 20% COD), kidney stone 2 (contains 70% CaHPO_4_ and 30% COM) and kidney stone 3 (contains 20% COM and 80% COD) from three different patients. (C) Extracted ion electropherograms of [^13^C_6_] Ins(1,2,3)P_3_ (black line) and InsP_3_ in kidney stone 1 (red area). (D) Extracted ion electropherograms of [^13^C_6_] Ins(1,2,3)P_3_ (black line) and InsP_3_ in kidney stone 2 (red area).

To investigate the presence and profiles of InsPs in kidney stones, different ground kidney stones of several compositions (calcium oxalate * *x*H_2_O (*x* = 1 COM, *x* = 2 COD) and calcium hydrogen phosphate (CaHPO_4_)), determined by IR spectroscopy, were extracted with perchloric acid according to the reported TiO_2_ purification method^51^ and then analyzed by CE-ESI-MS (Fig. 3A).^6^ We identify several InsPs as part of kidney stones providing the first such profiles (for a representative example see Fig. S4, ESI†). The high-resolution masses of the InsPs were confirmed by a CE-qTOF analysis (Fig. S5, ESI†). Some of the InsP isomers were identified with internal [^13^C_6_] labelled reference compounds. Fig. 3B summarizes the observed levels of different InsPs in calcium oxalate stones (kidney stone 1 contains 80% COM and 20% COD, kidney stone 3 contains 20% COM and 80% COD) and a calcium phosphate stone (kidney stone 2 contains 70% CaHPO_4_ and 30% COM), which roughly show the same trend in abundance of the analytes.

While inositol pyrophosphates were not detectable in these three types of calcium containing kidney stones, we noticed a decrease in InsP abundance proportional to the number of phosphate groups. InsP_6_ was the least abundant InsP followed by InsP_5_ and InsP_4_ isomers. One of the InsP_5_ isomers was assigned as 2-OH InsP_5_ (Fig. S4, ESI†) by spiking with an internal [^13^C_6_] reference. The most abundant InsP detected in kidney stone were InsP_2–3_ species and or isobaric sugar bisphosphates.

Full recovery for InsP_6–7_ and good recovery for 2-OH InsP_5_ from mammalian cell extracts were reported previously.^6^ Here our analysis shows good recovery for InsP_6_ (84%), 2-OH InsP_5_ (70%) and InsP_3_ (71%) from kidney stone extracts (kidney stone 2 is used as a representative example) by spiking with the [^13^C_6_] reference before extraction (pre-spiking) and after extraction but before measurement (post-spiking) (Fig. S6, ESI†). Since kidney stones are rich in Ca^2+^, which could affect InsP recovery, we reassessed TiO_2_ mediated InsP retrieval by adding 15 mM ethylenediaminetetraacetic acid (EDTA) during extraction. However, the recovery of InsP_6_ and InsP_3_ did not critically rely on presence or absence of additional EDTA.

Three peaks belonging to InsP_2_ and/or isobaric sugar bisphosphates were separated in kidney stone (Fig. S7, ESI†). Spiking indicated that none of these three peaks represents glucose-1,6-bisphosphate. The most intense peak has an identical migration time with [^13^C_6_] Ins(1/3,2)P_2_ generated by pyrohydrolysis from [^13^C_6_] Ins(1,2,3)P_3_ (Fig. S7, ESI†), indicating Ins(1,2)P_2_ and/or Ins(2,3)P_2_ are present in kidney stones. This is in line with recent findings that Ins(2,3)P_2_ is present in the μM range in immortalized mammalian cells.^27^

InsP_3_ was also comparably abundant in the calcium containing stones. Two peaks of InsP_3_ were recorded and identified as Ins(1,2,3)P_3_, representing the most intense peak, and Ins(1,2,6)P_3_ and/or its enantiomer Ins(2,3,4)P_3_. Identification was achieved by spiking with [^13^C_6_] Ins(1,2,3)P_3_ (Fig. 3C and D) and Ins(1,2,6)P_3_ (Fig. S8, ESI†). Ins(1,2,3)P_3_ was reported in mammalian cell models in a concentration range of 1–10 μM.^18^ A possible role for Ins(1,2,3)P_3_ as an intracellular iron chelator in the process of iron transport has been considered.^52,53^ Ins(1,2,6)P_3_ and/or the enantiomer Ins(2,3,4)P_3_ was proposed in mammalian B-cells in 1992,^54^ however, as discussed above, since then very few studies have been conducted to characterize their identity and functional roles and these isomers are missing in discussions in recent literature reviews.^21,22^

**Fig. 4:**
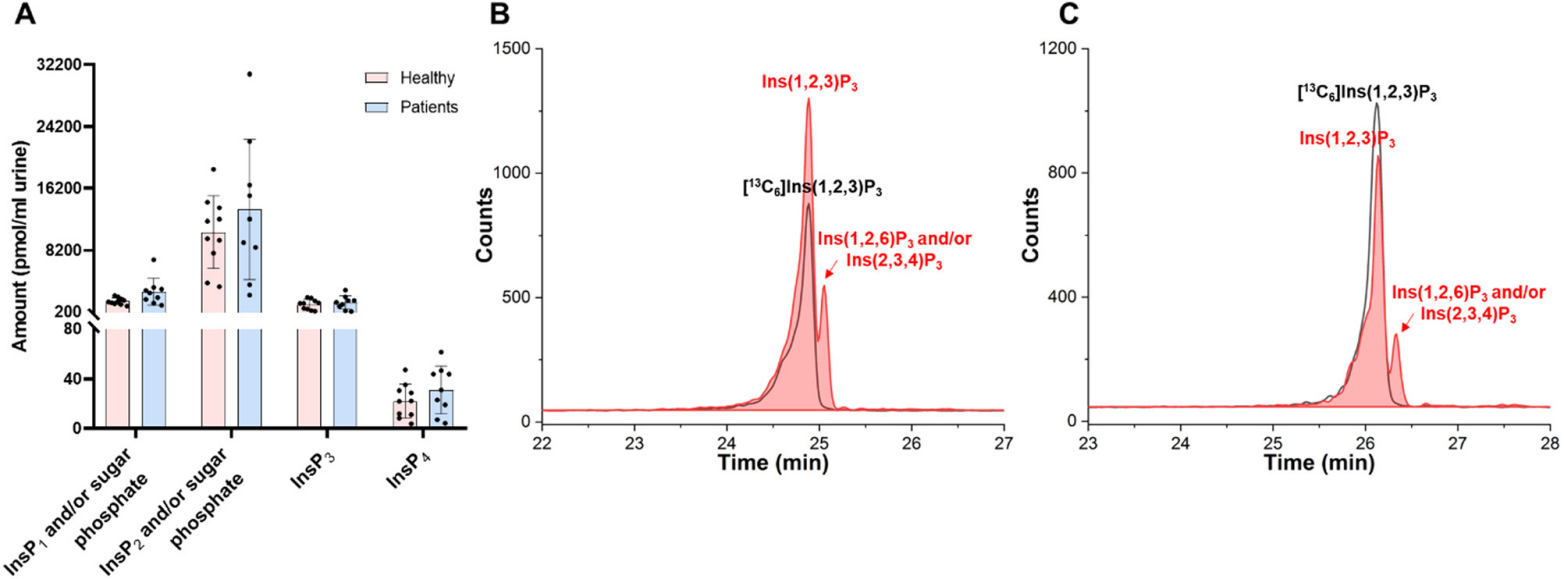
InsPs in urine samples from patients with kidney stones *vs.* healthy people. (A) InsPs distribution in urine samples: ten samples from healthy individuals, and nine samples from patients with kidney stones. (B) Extracted ion electropherograms of [^13^C_6_] Ins(1,2,3)P_3_ (black line) and InsP_3_ in urine from patients (red trace). (C) Extracted ion electropherograms of [^13^C_6_] Ins(1,2,3)P_3_ (black line) and InsP_3_ in urine from healthy individuals (red trace).

The formation of kidney stone is a result of urinary supersaturation and crystallization. To study potential correlations of the profiles of InsPs in kidney stones and urine, we additionally profiled InsPs in 0.4 mL urine samples both from patients who have kidney stones (9 urine samples from different donors) and matched healthy people (10 urine samples from different donors, Table S1, ESI†). The accurate masses of InsPs identified in urine samples were confirmed by a CE-qTOF analysis (for representative example see Fig. S9 and S10, ESI†). Since we cannot yet distinguish sugar mono and bisphosphates from InsP_1_ and InsP_2_ because of limitations of our current method, we must assume that *m*/*z* 259.0229 and *m*/*z* 338.9889 correspond to InsP_1_ and/or sugar phosphates and InsP_2_ and/or sugar bisphosphates, respectively. CE-QQQ was then used to profile InsP levels also in urine samples. The CE-QQQ results indicated that InsP_2_ and/or sugar bisphosphates are the most abundant species, followed by InsP_1_ and/or sugar phosphates. Interestingly, InsP_3_ was in the same concentration range as the sugar phosphates/InsP_1_ group of analytes and InsP_4_ was also present in the samples, but much less concentrated (*ca.* 7–8 fold) (Fig. 4A).

InsP_6_ was detectable in five of the urine samples from ten healthy people and only one of the urine samples from nine kidney stone patients. In all cases, the concentration of InsP_6_ was lower than the limit of quantification (LOQ). 2-OH InsP_5_ was also in between the LOD and LOQ in seven of the urine samples from ten healthy people and only three of the urine sample from nine kidney stone patients. According to the signal-to-noise of spiked 1 μM [^13^C_6_] InsP_6_ and [^13^C_6_] 2-OH InsP_5_, we determined limits of detection (LOD) of InsP_6_ and 2-OH InsP_5_ in the urine samples analyzed were approx. 7.5 nM and 1.5 nM InsP_5_, respectively. Limits of quantitation (LOQ) of InsP_6_ and 2-OH InsP_5_ were approx. 22.5 nM and 4.5 nM, respectively. Therefore, less than 7.5 nM of InsP_6_ exist in most of these urine samples, which is in accordance with the reported detection using InsPs specific assays.^51,55,56^ In half of these samples, approx. 1.5 nM to 4.5 nM 2-OH InsP_5_ exist. InsP_4_ were also present in urine samples. InsP_5_ and InsP_4_ received little attention so far in human urine, and our results provide an initial overview of these species. A study of InsPs in rat urine after giving InsP_6_ dietary treatment indicated that InsP_6_ as well InsP_2_, InsP_3_, InsP_4_ and InsP_5_ is excreted in the urine.^55^ The source of those isomers remains obscure.

Similar to the results obtained for kidney stones, InsP_2_ and/or sugar bisphosphates are most abundant. The most intense species comigrates with [^13^C_6_] Ins(1/3,2)P_2_ generated by pyrohydrolysis of [^13^C_6_] Ins(1,2,3)P_3_ (Fig. S9, ESI†). Also in agreement with the results found in kidney stones, two peaks corresponding to the mass of InsP_3_ in urine samples can be identified as Ins(1,2,3)P_3_ and Ins(1,2,6)P_3_ and/or the enantiomer Ins(2,3,4)P_3_ (Fig. 4B, C and Fig. S11, ESI†). According to our current data set, there is no significant difference of InsPs and/or sugar phosphates in urine from patients and healthy people regarding levels of InsP_1_ to InsP_4_. For InsP_5–6_ the picture is less clear as the measured concentrations were in between our current LOD and LOQ for most samples.

## Conclusions

We have developed a CE-MS method to separate “lower” InsPs, particularly focusing on InsP_2_ and InsP_3_. Using this method combined with a pyrohydrolysis strategy, we were able to delineate the identity of a major human InsP_3_, *i.e.* Ins(1,2,3)P_3_ in urine and kidney stones. To achieve this goal, a phytase (RipBL1) was used that selectively degrades InsP_6_ to Ins(1,2,3)P_3_ and that also served for production of a ^13^C labelled Ins(1,2,3)P_3_ internal reference. Our profiling of InsPs in human kidney stone and urine also unveiled Ins(1,2,6)P_3_ and/or the enantiomer Ins(2,3,4)P_3_ besides the major Ins(1,2,3)P_3_. Importantly, the assignments of InsP_3_ are based on an accurate mass determination and comigration with standards. As a next step, further studies must delineate the stereoisomeric identity of the newly identified InsP_3_, since chiral selectors have already been developed for certain InsP enantiomers for assignments by ^31^P NMR spectroscopy.^32^ Additionally, methods to distinguish InsP_1_ and InsP_2_ from sugar (bis)phosphates will have to be developed. Derivatization of sugar phosphates to separate them from InsPs could be considered.^57^ Further improvements in LOQs will be helpful to establish, if potentially InsP_5_ or InsP_6_ concentrations in urine can be used as biomarkers for kidney stone formation.

Ins(1,2,3)P_3_ was described in mammalian cells more than 25 years ago^18,26^ and other inositol phosphates with a phosphate ester in the 2-position clearly exist. However, the interest in these isomers in human biology has faded over time, and recent literature is not reporting on them anymore so their roles remain unresolved. Our study in combination with work from the Fiedler group^27^ demonstrates that Ins(2)Ps are abundant cellular, kidney stone and body fluid components. These discoveries warrant reassessment of the classically discussed InsP metabolism. New attention must be given to noncanonical InsP species to fully and properly appreciate the inositol phosphate code.

## Author contributions

G. L., A. S., and H. J. J. designed research and wrote the paper. G. L., D. Q. and H. J. J. analyzed data, E. R., R. S., G. S., A. S., T. L. cloned and analyzed RipBL1, D. C., O. B., C. A. W., J. P. J., T. K. provided materials and analyzed data. All authors discussed the paper and provided input for the final version. Further Swiss Kidney Stone Cohort investigators are listed in the acknowledgments.

## Conflicts of interest

The authors declare no conflicts of interest.

## Supplementary Material

CB-004-D2CB00235C-s001

## References

[cit1] York J. D. (2006). Regulation of nuclear processes by inositol polyphosphates. Biochim. Biophys. Acta.

[cit2] Irvine R. F., Schell M. J. (2001). Back in the water: the return of the inositol phosphates. Nat. Rev. Mol. Cell Biol..

[cit3] Otto J. C., Kelly P., Chiou S. T., York J. D. (2007). Alterations in an inositol phosphate code through synergistic activation of a G protein and inositol phosphate kinases. Proc. Natl. Acad. Sci. U. S. A..

[cit4] Dovey C. M., Diep J., Clarke B. P., Hale A. T., McNamara D. E., Guo H., Brown, Jr. N. W., Cao J. Y., Grace C. R., Gough P. J., Bertin J., Dixon S. J., Fiedler D., Mocarski E. S., Kaiser W. J., Moldoveanu T., York J. D., Carette J. E. (2018). MLKL Requires the Inositol Phosphate Code to Execute Necroptosis. Mol. Cell.

[cit5] Ito M., Fujii N., Wittwer C., Sasaki A., Tanaka M., Bittner T., Jessen H. J., Saiardi A., Takizawa S., Nagata E. (2018). Hydrophilic interaction liquid chromatography-tandem mass spectrometry for the quantitative analysis of mammalian-derived inositol poly/pyrophosphates. J. Chromatogr. A.

[cit6] Qiu D., Wilson M. S., Eisenbeis V. B., Harmel R. K., Riemer E., Haas T. M., Wittwer C., Jork N., Gu C., Shears S. B., Schaaf G., Kammerer B., Fiedler D., Saiardi A., Jessen H. J. (2020). Analysis of inositol phosphate metabolism by capillary electrophoresis electrospray ionization mass spectrometry. Nat. Commun..

[cit7] Wilson M. S. C., Saiardi A. (2017). Importance of Radioactive Labelling to Elucidate Inositol Polyphosphate Signalling. Top Curr Chem (Cham).

[cit8] Sprigg C., Whitfield H., Burton E., Scholey D., Bedford M. R., Brearley C. A. (2022). Phytase dose-dependent response of kidney inositol phosphate levels in poultry. PLoS One.

[cit9] Li P., Gawaz M., Chatterjee M., Lämmerhofer M. (2021). Isomer-selective analysis of inositol phosphates with differential isotope labelling by phosphate methylation using liquid chromatography with tandem mass spectrometry. Anal. Chim. Acta.

[cit10] Lin H., Fridy P. C., Ribeiro A. A., Choi J. H., Barma D. K., Vogel G., Falck J. R., Shears S. B., York J. D., Mayr G. W. (2009). Structural analysis and detection of biological inositol pyrophosphates reveal that the family of VIP/diphosphoinositol pentakisphosphate kinases are 1/3-kinases. J. Biol. Chem..

[cit11] Albert C., Safrany S. T., Bembenek M. E., Reddy K. M., Reddy K., Falck J., Brocker M., Shears S. B., Mayr G. W. (1997). Biological variability in the structures of diphosphoinositol polyphosphates in Dictyostelium discoideum and mammalian cells. Biochem J..

[cit12] Buscher B. A. P., Vanderhoeven R. A. M., Tjaden U. R., Andersson E., Vandergreef J. (1995). Analysis of Inositol Phosphates and Derivatives Using Capillary Zone Electrophoresis Mass-Spectrometry. J. Chromatogr. A.

[cit13] Buscher B. A. P., Hofte A. J. P., Tjaden U. R., vanderGreef J. (1997). On-line electrodialysis capillary zone electrophoresis mass spectrometry of inositol phosphates in complex matrices. J. Chromatogr. A.

[cit14] Riemer E., Qiu D., Laha D., Harmel R. K., Gaugler P., Gaugler V., Frei M., Hajirezaei M. R., Laha N. P., Krusenbaum L., Schneider R., Saiardi A., Fiedler D., Jessen H. J., Schaaf G., Giehl R. F. H. (2021). ITPK1 is an InsP6/ADP phosphotransferase that controls phosphate signaling in Arabidopsis. Mol. Plant.

[cit15] Qiu D., Eisenbeis V. B., Saiardi A., Jessen H. J. (2021). Absolute Quantitation of Inositol Pyrophosphates by Capillary Electrophoresis Electrospray Ionization Mass Spectrometry. J. Vis Exp..

[cit16] Qiu D., Gu C., Liu G., Ritter K., Eisenbeis V. B., Bittner T., Gruzdev A., Seidel L., Bengsch B., Shears S. B., Jessen H. J. (2023). Capillary electrophoresis mass spectrometry identifies new isomers of inositol pyrophosphates in mammalian tissues. Chem. Sci..

[cit17] Thomas M. P., Mills S. J., Potter B. V. (2016). The “Other” Inositols and Their Phosphates: Synthesis, Biology, and Medicine (with Recent Advances in myo-Inositol Chemistry). Angew. Chem., Int. Ed..

[cit18] Barker C. J., French P. J., Moore A. J., Nilsson T., Berggren P. O., Bunce C. M., Kirk C. J., Michell R. H. (1995). Inositol 1,2,3-trisphosphate and inositol 1,2- and/or 2,3-bisphosphate are normal constituents of mammalian cells. Biochem J..

[cit19] Chen Q. C., Li B. W. (2003). Separation of phytic acid and other related inositol phosphates by high-performance ion chromatography and its applications. J. Chromatogr. A.

[cit20] Streb H., Irvine R. F., Berridge M. J., Schulz I. (1983). Release of Ca2+ from a nonmitochondrial intracellular store in pancreatic acinar cells by inositol-1,4,5-trisphosphate. Nature.

[cit21] Lee B., Park S. J., Hong S., Kim K., Kim S. (2021). Inositol Polyphosphate Multikinase Signaling: Multifaceted Functions in Health and Disease. Mol. Cells.

[cit22] Chatree S., Thongmaen N., Tantivejkul K., Sitticharoon C., Vucenik I. (2020). Role of Inositols and Inositol Phosphates in Energy Metabolism. Molecules.

[cit23] Shears S. B. (2020). A Short Historical Perspective of Methods in Inositol Phosphate Research. Methods Mol. Biol..

[cit24] Hale A. T., Clarke B. P., York J. D. (2020). Metabolic Labeling of Inositol Phosphates and Phosphatidylinositols in Yeast and Mammalian Cells. Methods Mol. Biol..

[cit25] Barker C. J., Wright J., Hughes P. J., Kirk C. J., Michell R. H. (2004). Complex changes in cellular inositol phosphate complement accompany transit through the cell cycle. Biochem J.

[cit26] Barker C. J., Wright J., Kirk C. J., Michell R. H. (1995). Inositol 1,2,3-trisphosphate is a product of InsP6 dephosphorylation in WRK-1 rat mammary epithelial cells and exhibits transient concentration changes during the cell cycle. Biochem. Soc. Trans..

[cit27] Nguyen Trung M., Kieninger S., Fandi Z., Qiu D., Liu G., Mehendale N. K., Saiardi A., Jessen H., Keller B., Fiedler D. (2022). Stable Isotopomers of myo-Inositol Uncover a Complex MINPP1-Dependent Inositol Phosphate Network. ACS Cent. Sci..

[cit28] Wilson M. S., Jessen H. J., Saiardi A. (2019). The inositol hexakisphosphate kinases IP6K1 and -2 regulate human cellular phosphate homeostasis, including XPR1-mediated phosphate export. J. Biol. Chem..

[cit29] Moritoh Y., Abe S. I., Akiyama H., Kobayashi A., Koyama R., Hara R., Kasai S., Watanabe M. (2021). The enzymatic activity of inositol hexakisphosphate kinase controls circulating phosphate in mammals. Nat. Commun..

[cit30] Li X., Gu C., Hostachy S., Sahu S., Wittwer C., Jessen H. J., Fiedler D., Wang H., Shears S. B. (2020). Control of XPR1-dependent cellular phosphate efflux by InsP8 is an exemplar for functionally-exclusive inositol pyrophosphate signaling. Proc. Natl. Acad. Sci. U. S. A..

[cit31] Lopez-Sanchez U., Tury S., Nicolas G., Wilson M. S., Jurici S., Ayrignac X., Courgnaud V., Saiardi A., Sitbon M., Battini J. L. (2020). Interplay between primary familial brain calcification-associated SLC20A2 and XPR1 phosphate transporters requires inositol polyphosphates for control of cellular phosphate homeostasis. J. Biol. Chem..

[cit32] Bluher D., Laha D., Thieme S., Hofer A., Eschen-Lippold L., Masch A., Balcke G., Pavlovic I., Nagel O., Schonsky A., Hinkelmann R., Worner J., Parvin N., Greiner R., Weber S., Tissier A., Schutkowski M., Lee J., Jessen H., Schaaf G., Bonas U. (2017). A 1-phytase type III effector interferes with plant hormone signaling. Nat. Commun..

[cit33] Puschmann R., Harmel R. K., Fiedler D. (2019). Scalable Chemoenzymatic Synthesis of Inositol Pyrophosphates. Biochemistry.

[cit34] Harmel R. K., Puschmann R., Nguyen Trung M., Saiardi A., Schmieder P., Fiedler D. (2019). Harnessing (13)C-labeled myo-inositol to interrogate inositol phosphate messengers by NMR. Chem. Sci..

[cit35] Marolt G., Kolar M. (2020). Analytical Methods for Determination of Phytic Acid and Other Inositol Phosphates: A Review. Molecules.

[cit36] Cosgrove D. J. (1969). Ion-exchange chromatography of inositol polyphosphates. Ann. N. Y. Acad. Sci..

[cit37] Lim P. E., Tate M. E. (1973). The phytases. II. Properties of phytase fractions F 1 and F 2 from wheat bran and the myoinositol phosphates produced by fraction F 2. Biochim. Biophys. Acta.

[cit38] Barrientos L., Scott J. J., Murthy P. P. (1994). Specificity of hydrolysis of phytic acid by alkaline phytase from lily pollen. Plant Physiol..

[cit39] Freund W. D., Mayr G. W., Tietz C., Schultz J. E. (1992). Metabolism of inositol phosphates in the protozoan Paramecium. Characterization of a novel inositol-hexakisphosphate-dephosphorylating enzyme. Eur. J. Biochem..

[cit40] Van der Kaay J., Van Haastert P. J. (1995). Stereospecificity of inositol hexakisphosphate dephosphorylation
by Paramecium phytase. Biochem J..

[cit41] Nakano T., Joh T., Narita K., Hayakawa T. (2000). The pathway of dephosphorylation of myo-inositol hexakisphosphate by phytases from wheat bran of Triticum aestivum L. cv. Nourin #61. Biosci., Biotechnol., Biochem..

[cit42] Brearley C. A., Hanke D. E. (1996). Inositol phosphates in barley (Hordeum vulgare L.) aleurone tissue are stereochemically similar to the products of breakdown of InsP6 in vitro by wheat-bran phytase. Biochem J..

[cit43] Alelign T., Petros B. (2018). Kidney Stone Disease: An Update on Current Concepts. Adv. Urol..

[cit44] Wang Z., Zhang Y., Zhang J., Deng Q., Liang H. (2021). Recent advances on the mechanisms of kidney stone formation (Review. Int. J. Mol. Med..

[cit45] Fakier S., Rodgers A., Jackson G. (2019). Potential thermodynamic and kinetic roles of phytate as an inhibitor of kidney stone formation: theoretical modelling and crystallization experiments. Urolithiasis.

[cit46] Saw N. K., Chow K., Rao P. N., Kavanagh J. P. (2007). Effects of inositol hexaphosphate (phytate) on calcium binding, calcium oxalate crystallization and in vitro stone growth. J. Urol..

[cit47] Grases F., Costa-Bauza A., March J. G. (1994). Artificial simulation of the early stages of renal stone formation. Br. J. Urol..

[cit48] Schantl A. E., Verhulst A., Neven E., Behets G. J., D'Haese P. C., Maillard M., Mordasini D., Phan O., Burnier M., Spaggiari D., Decosterd L. A., MacAskill M. G., Alcaide-Corral C. J., Tavares A. A. S., Newby D. E., Beindl V. C., Maj R., Labarre A., Hegde C., Castagner B., Ivarsson M. E., Leroux J. C. (2020). Inhibition of vascular calcification by inositol phosphates derivatized with ethylene glycol oligomers. Nat. Commun..

[cit49] Kletzmayr A., Mulay S. R., Motrapu M., Luo Z., Anders H. J., Ivarsson M. E., Leroux J. C. (2020). Inhibitors of Calcium Oxalate Crystallization for the Treatment of Oxalate Nephropathies. Adv. Sci..

[cit50] Sinha S., Gould L. J., Nigwekar S. U., Serena T. E., Brandenburg V., Moe S. M., Aronoff G., Chatoth D. K., Hymes J. L., Miller S., Padgett C., Carroll K. J., Perello J., Gold A., Chertow G. M. (2022). The CALCIPHYX study: a randomized, double-blind, placebo-controlled, Phase 3 clinical trial of SNF472 for the treatment of calciphylaxis. Clin Kidney J..

[cit51] Wilson M. S., Bulley S. J., Pisani F., Irvine R. F., Saiardi A. (2015). A novel method for the purification of inositol phosphates from biological samples reveals that no phytate is present in human plasma or urine. Open Biol..

[cit52] Sala M., Makuc D., Kolar J., Plavec J., Pihlar B. (2011). Potentiometric and (3)(1)P NMR studies on inositol phosphates and their interaction with iron(III) ions. Carbohydr. Res..

[cit53] Veiga N., Torres J., Mansell D., Freeman S., Dominguez S., Barker C. J., Diaz A., Kremer C. (2009). “Chelatable iron pool”: inositol 1,2,3-trisphosphate fulfils the conditions required to be a safe cellular iron ligand. J. Biol. Inorg. Chem..

[cit54] McConnell F. M., Shears S. B., Lane P. J., Scheibel M. S., Clark E. A. (1992). Relationships between the degree of cross-linking of surface immunoglobulin and the associated inositol 1,4,5-trisphosphate and Ca2+ signals in human B cells. Biochem J..

[cit55] Grases F., Costa-Bauza A., Berga F., Rodriguez A., Gomila R. M., Martorell G., Martinez-Cignoni M. R. (2018). Evaluation of inositol phosphates in urine after topical administration of myo-inositol hexaphosphate to female Wistar rats. Life Sci..

[cit56] Letcher A. J., Schell M. J., Irvine R. F. (2008). Do mammals make all their own inositol hexakisphosphate. Biochem. J..

[cit57] Rende U., Niittyla T., Moritz T. (2019). Two-step derivatization for determination of sugar phosphates in plants by combined reversed phase chromatography/tandem mass spectrometry. Plant Methods.

