# Supplementary material for: The phytase RipBL1 enables the assignment of a specific inositol phosphate isomer as a structural component of human kidney stones
Source: RSC Chem Biol. 2023 Jan 27;4(4):300–9. doi: 10.1039/d2cb00235c (PMC10074554; doi:10.1039/d2cb00235c)
Supplement: CB-004-D2CB00235C-s001 [file CB-004-D2CB00235C-s001.pdf]

Electronic Supplementary Material (ESI) for RSC Chemical Biology.
This journal is © The Royal Society of Chemistry 2023

Supplementary Information

The phytase RipBL1 enables the assignment of a specific inositol phosphate isomer as a structural component of human kidney stones

Guizhen Liu[1], Esther Riemer[2], Robin Schneider[2], Daniela Cabuzu[3], Olivier Bonny[3], Carsten A. Wagner[4], Danye Qiu[1], Adolfo Saiardi[5], Annett Strauss[6], Thomas Lahaye[6], Gabriel Schaaf[2], Thomas Knoll[7], Jan P. Jessen[7], and Henning J. Jessen[1*]

[1]Institute of Organic Chemistry & CIBSS – Centre for Integrative Biological Signalling Studies, University of Freiburg, Germany

[2]Institute of Crop Science and Resource Conservation, Department of Plant Nutrition, University of Bonn, Germany

[3] Department of Biomedical Sciences, University of Lausanne, Switzerland and Service of Nephrology, Lausanne University Hospital, Lausanne, Switzerland. Present address: Service of nephrology, Fribourg State Hospital, Fribourg, Switzerland

[4] Institute of Physiology, University of Zürich, Switzerland

[5] Laboratory for Molecular Cell Biology, University College London, United Kingdom

[6] ZMBP, University of Tübingen, Germany

[7] Department of Urology, Sindelfingen-Boeblingen Medical Center, Teaching Hospital University Tübingen, Sindelfingen, Germany

- List of SKSC investigators: Harald Seeger and Alexander Ritter, University Hospital Zurich; Olivier Bonny, Gregoire Wuerzner and Beat Roth, University Hospital Lausanne; Daniel Fuster and Nasser Dahyat, University Hospital Bern; Florian Buchkremer and Stephan Segerer, Kantonspital Aarau; Thomas Ernandez and Catherine Stoermann-Chopard, University Hospital Geneva; Carsten A. Wagner, University of Zurich.

Supplementary Methods

RipBL1 expression and purification

For expression, an overnight culture of *Escherichia coli* BL21-CodonPlus (DE3)-RIL cells (Stratagene, La Jolla, CA, USA) harboring pDest527-RipBL1 was inoculated in 2YT containing 100 µg/ml ampicillin and 25 µg/ml chloramphenicol. At OD_{600} = 0.8, the culture was cooled on ice and protein expression was induced by 400 µM IPTG and continued shaking at 16°C for 22 hours.

To purify RipBL1, the cell pellet was resuspended in 300 mM NaCl, 25 mM Na_2HPO_4, pH 7.5, 2 mM DTT, 1 mM EDTA and 1 mg/ml lysozyme. After 30 min at 4°C, rotating, cells were disrupted by vortexing for 1 min with glass beads, followed by incubation on ice for 1 min, each step repeated eight times. The cell lysate was collected by centrifugation at 2800 g for 15 min, followed by centrifugation at 18000 g for 45 min at 4°C, respectively. The lysate was incubated with Ni-NTA-resin (Protino) overnight and then washed in 300 mM NaCl, 25 mM Na_2HPO_4, pH 7.5, 25 mM imidazole and 1 mM DTT. The protein was eluted in the same buffer but with 250 mM imidazole. Protein purity and concentration of dialysed RipBL1 was checked by SDS-PAGE and Coomassie staining and BSA standards.

$InsP_6$ degradation assay by RipBL1

0.14 µg/µl recombinant RipBL1 was incubated in a reaction mixture of 50 mM HEPES, 10 mM NaCl, 5% glycerol, 2 mM DTT, 0.5 mM $MgCl_2$, pH 7.0, and 5 mM $InsP_6$ (Sichem) or 0.33 mM [$^{13}C_6$] $InsP_6$, respectively. The assay was conducted at 28°C for 45 min.

Extraction of PP-InsPs and InsPs from kidney stone and urine

Urine samples were obtained from fresh void of the second morning urine and immediately frozen at -80°C upon further processing. Nine kidney stone formers and 10 controls free of stone (no kidney stone history and no detectable calcification on an abdominal CT-scan) from the Swiss Kidney Stone Cohort (SKSC) did participate, the characteristic of the participants are shown in Table S1. SKSC is registered at ClinicalTrial.gov under the identifier NCT01990027 and was approved by the Swiss ethic committee.

Table S1. Characteristics of the participants

Items	Controls	Stone formers
N	10	9
Mean age (yrs)	52,2	50,1
Female/male (n)	5/5	5/4

Mean number of stone events		0	2,67
Stone composition (number of components)			
	Calcium oxalate monohydrate	-	4
	Calcium oxalate dihydrate	-	3
	Uric acid	-	2
	Apatite	-	1
	No analysis available	-	3
Medication at the time of urine sampling			
	Anti-hypertensive (ARA, HCT, amlodipine)	3	1
	Citrate salts (potassium or magnesium)		4
	Anti-diabetics (metformin and SGLT2i)		4
	Proton pump inhibitors		1
	Bisphosphonate		1
	Acenocoumarol		1
	Statine		1
	Fluoxetine	1	
	Levothyroxine	1	

Extraction of kidney stones: kidney stones (composition determined by IR spectroscopy) were powdered with a grinder, then 10 mg powder was weighted and homogenized in 1 ml of ice-cold perchloric acid (1M), incubated at 4°C with rotation for 20 minutes. The homogenate was centrifuged with max speed. The supernatant was transferred to pre-washed titanium dioxide (TiO_2) beads for InsPs purification.[1] Briefly, the supernatant was incubated with 5 mg TiO_2 beads at 4°C for 20 minutes to capture inositol phosphates. The beads were washed with 1M perchloric acid solution and then the inositol phosphates eluted with 3% ammonium hydroxide. The elution solution was dried in a speed vac evaporator.

Extraction of urine: centrifuge 0.4 ml urine at 2000*g* at 4°C for 5 minutes. Transfer the supernatant to 0.4 ml of 2 M perchloric acid (ice-cold) and rotate at 4°C for 20 minutes. The homogenate was centrifuged with max speed. Transfer the supernatant to 10 mg of pre-washed titanium dioxide (TiO_2) beads for InsPs purification as described above.

Pyrohydrolysis of InsPs

15 µg/ml of Ins(3,4,5)P_3, 16 µg/ml of Ins(2,3,5,6)P_4, 5 µg/ml of Ins(1,4,5,6)P_4 and 38.7 µM Ins(1,2,3)P_3 InsPs solution were incubated at 100°C for 2.5 h, respectively. InsPs solutions were prepared with ultrapure water, no further pH adjustment was done.

CE-QQQ analysis

All kidney stone extract samples were dissolved in 50 µl MilliQ water, and all urine extracts were dissolved in 30 µl MilliQ water. Quantitation of $InsP_5$ and $InsP_6$ were performed with

known amounts of isotopic standards spiked as internal standards. Quantitation of $InsP_1$, $InsP_2$, and $InsP_4$ of which no isotopic standards are available was performed with spiked [$^{13}C_6$] $InsP_6$. In order to compare with $InsP_1$, $InsP_2$ and $InsP_4$, quantitation of $InsP_3$ was also performed with spiked [$^{13}C_6$] $InsP_6$ for comparison, although [$^{13}C_6$] Ins (1,2,3) P_3 is available in this study. After spiking, 20 μM [$^{13}C_6$] 2-OH $InsP_5$, 20 μM [$^{13}C_6$] $InsP_6$, 4 μM [$^{13}C_6$] 5-$InsP_7$, 2 μM [$^{13}C_6$] 1-$InsP_7$ and 1 μM [$^{13}C_6$] 1,5-$InsP_8$ were the final concentration inside samples.

The analysis was achieved with a CE-ESI-QQQ system (Agilent 7100 CE with Agilent 6495C Triple Quadrupole and Agilent Jet Stream electrospray ionization source, adopting an Agilent CE-ESI-MS interface). MS source parameters setting and MRM transitions setting are shown below. An isocratic LC pump was used to constantly deliver the sheath liquid (water: isopropanol=1:1, v/v) via a splitter with a ratio of 1:100, the final splitting flow was set to 10 μl/min. A bare fused silica capillary with a length of 100 cm (50 μm internal diameter and 365 μm outer diameter) was used for CE separation. Two background electrolytes (BGE) were employed, one is 35 mM ammonium acetate titrated with ammonium hydroxide to pH 9.75, the other one is 50 mM ethylamine titrated with formic acid to pH 10.0. Before running, the capillary was flushed with BGE for 400 s. 20 nl of sample was injected by applying pressure (100 mbar for 10 s).

**Source Parameters**	
Gas Temperature	150 °C
Gas Flow	11 L/min
Nebulizer	8 psi
Sheath Gas Temperature	175 °C
Sheath Gas Flow	8 L/min
Capillary Voltage	-2000 V
Nozzle Voltage	2000 V
High Pressure RF (Ion Funnel Parameters)	70 V
Low Pressure RF (Ion Funnel Parameters)	40 V

**Molecular name**	**Precursor Ion**	**Product Ion**	**dwell**	**Fragmentor (V)**	**Collision Energy (V)**	**Cell Accelerator**	**Polarity**

						**Voltage**	
$InsP_1$	259	79.1	50	166	41	4	Negative
$InsP_2$	339	241	50	166	21	4	Negative
$InsP_3$	418.9	320.8	50	166	17	4	Negative
$[^{13}C_6]$ $InsP_3$	424.9	326.8	50	166	17	4	Negative
$InsP_4$	249	418.9	50	166	5	1	Negative
$InsP_5$	289	498.9	50	166	9	3	Negative
$[^{13}C_6]$ $InsP_5$	292	504.9	50	166	9	3	Negative
$InsP_6$	328.9	480.9	50	166	13	4	Negative
$[^{13}C_6]$ $InsP_6$	331.9	486.9	50	166	13	4	Negative
$InsP_7$	368.9	319.9	50	166	9	3	Negative
$[^{13}C_6]$ $InsP_7$	371.9	322.9	50	166	9	3	Negative
$InsP_8$	408.9	359.8	50	166	9	1	Negative
$[^{13}C_6]$ $InsP_8$	411.9	362.8	50	166	9	1	Negative

Supporting Figures

	Range (µg/ml)	Linear equation (n=3)	R^2	LOD (µg/ml, n=3)	LOQ (µg/ml, n=3)
Ins(1,2)P_2	0.1-10	y = 9447.3x - 409.54	0.9982	0.025	0.05
Ins(1,2,6)P_3	0.4-40	y = 5122.6x - 1267	0.9973	0.020	0.10

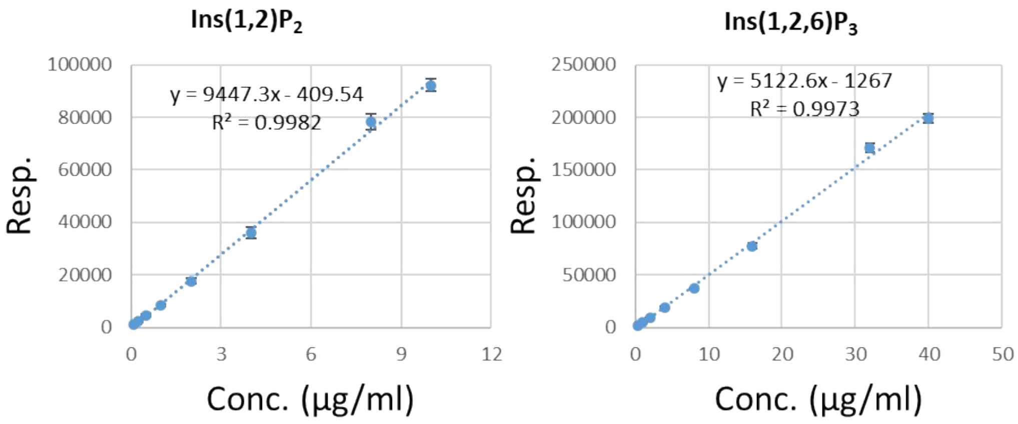

Figure S1. Calibration curves for quantitation of Ins (1,2) P_2 and Ins (1,2,6) P_3 with standards. Area of peaks are plotted against the concentration of standards. A signal-to-noise ratio between 3 to 10 is considered acceptable for the LOD, and a signal-to-noise ratio above 10 is considered acceptable for the LOQ.

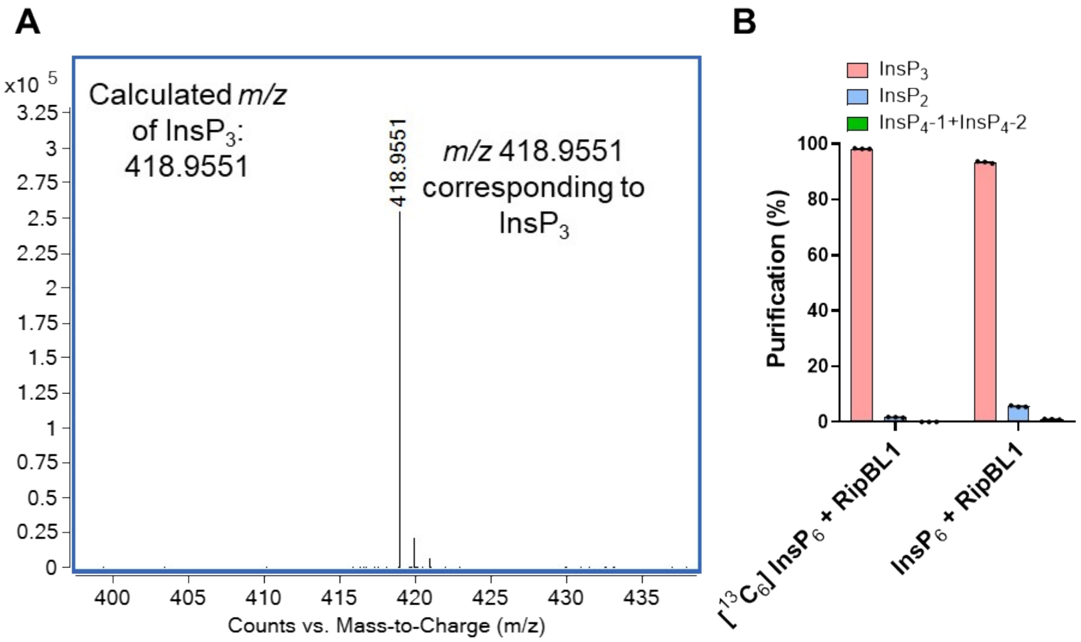

Figure S2. A CE-QTOF-MS of the dephosphorylation product of $InsP_6$ after treatment with RipBL1 enzyme. The *m/z* 418.9551 corresponds to $InsP_3$. **B** Distribution of dephosphorylation products of [$^{13}C_6$] $InsP_6$ and $InsP_6$ by RipBL1.

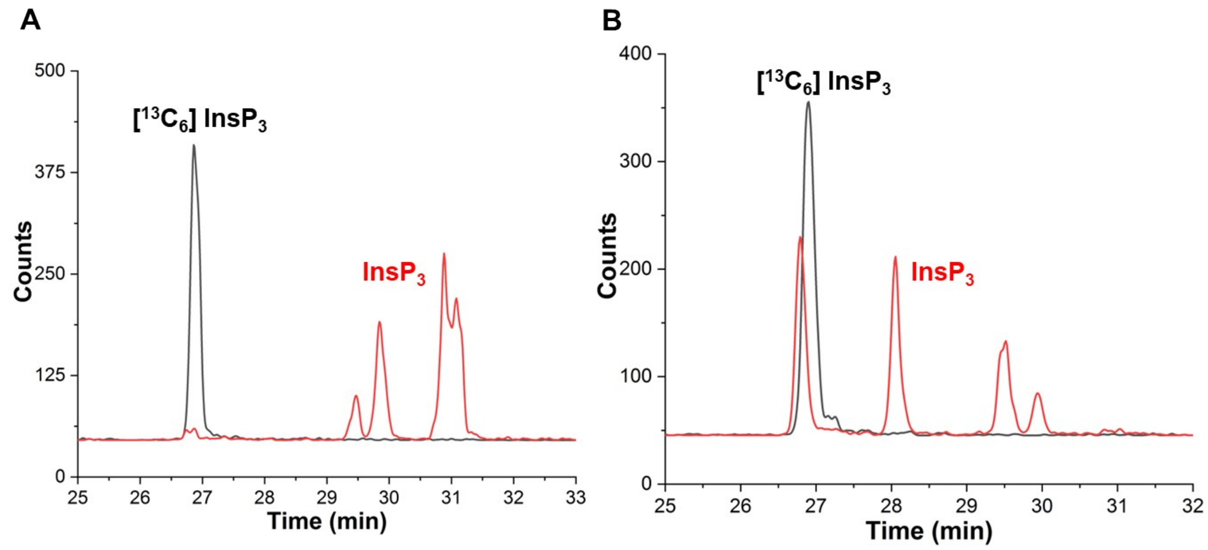

Figure S3 [$^{13}C_6$] $InsP_3$ spiked with pyrohydrolysis products of Ins (2,3,5,6) P_4 and Ins (1,4,5,6) P_4 solution after heating to 100°C for 2.5 h. **A.** Extracted ion electropherograms of [$^{13}C_6$] $InsP_3$ (black lines) plus $InsP_3$ (red line) generated by Ins (2,3,5,6) P_4. The [$^{13}C_6$] $InsP_3$ is not any of the $InsP_3$ generated from Ins (2,3,5,6) P_4 because of inconsistent migration times. **B.** Extracted ion electropherograms of [$^{13}C_6$] $InsP_3$ (black lines) plus $InsP_3$ (red line) generated by Ins (1,4,5,6) P_4. The [$^{13}C_6$] $InsP_3$ is not any of the $InsP_3$ generated from Ins (1,4,5,6) P_4 because of inconsistent migration times.

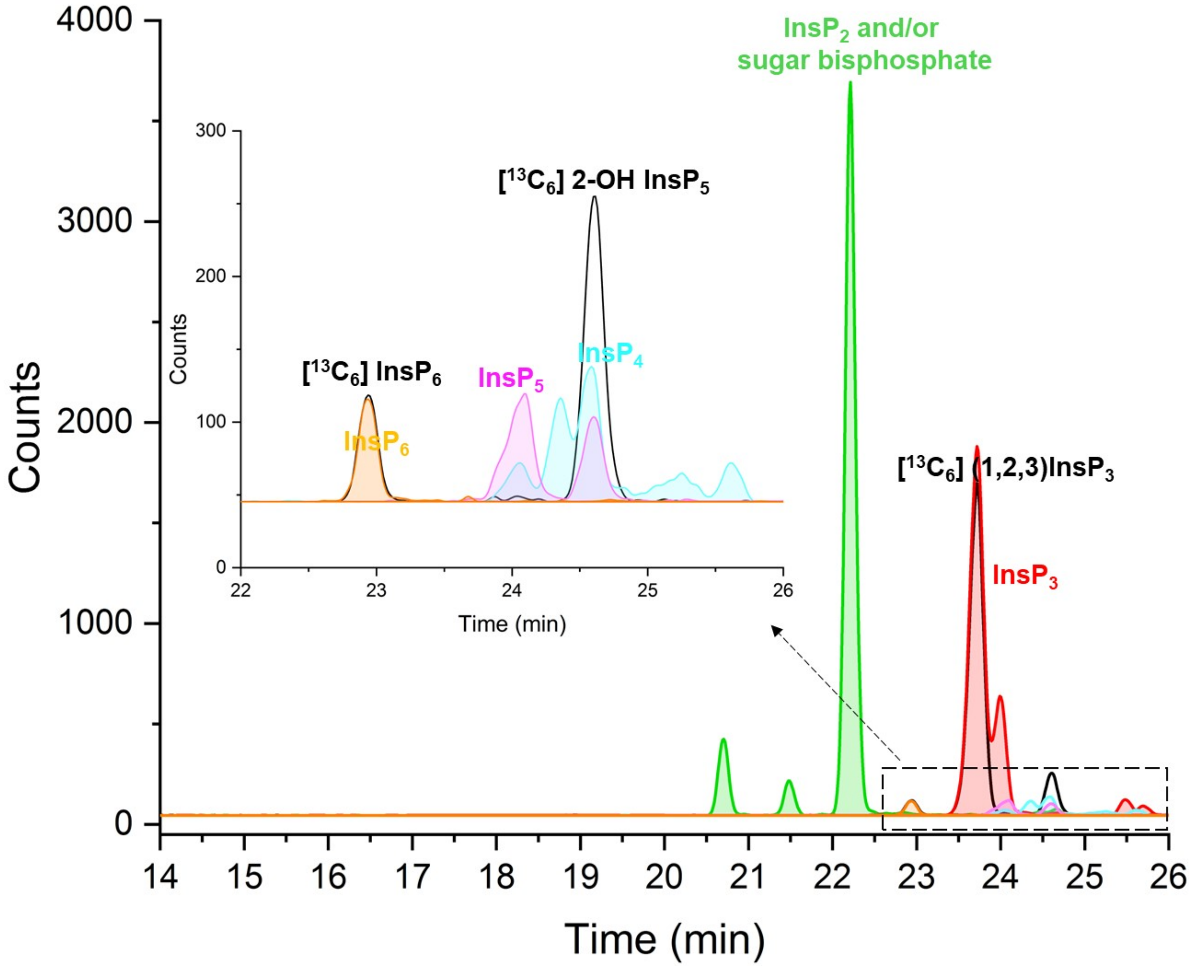

Figure S4 Extracted ion electropherograms of InsPs in kidney stone by CE-QQQ. The InsPs were assigned by identical migration time with spiked [$^{13}C_6$] internal standards. Accurate masses of these InsPs and/or sugar bisphosphate were confirmed by CE-qTOF. [$^{13}C_6$] InsPs: black solid line, $InsP_6$: gold trace, $InsP_5$: purple trace, $InsP_4$: light blue trace, $InsP_3$: red trace, $InsP_2$ and /or sugar bisphosphate: green trace.

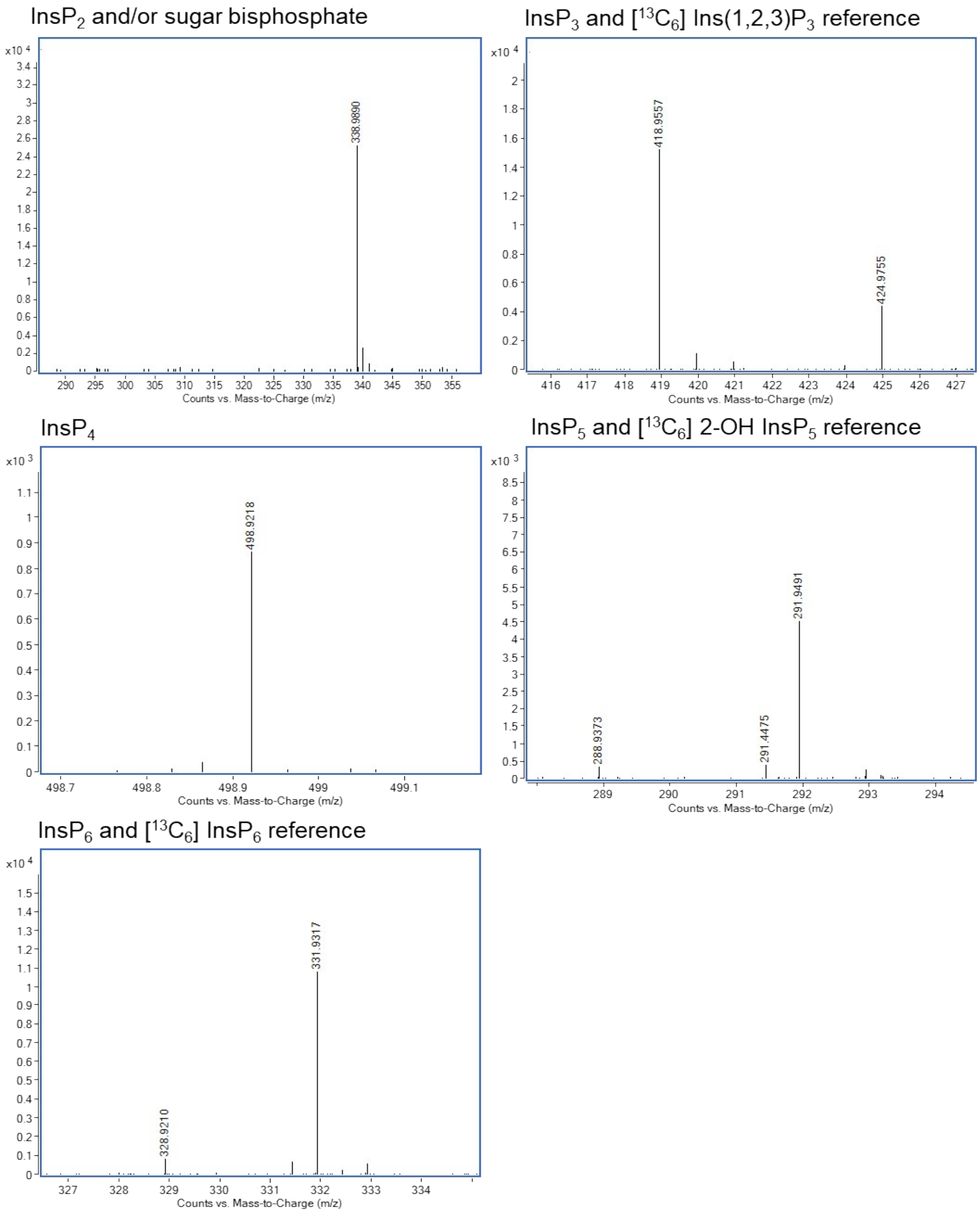

Figure S5 High-resolution mass spectra of InsPs from CE-qTOF analysis of kidney stone spiked with [$^{13}C_6$] InsP references. Theoretical mass to charge value for $InsP_2$ or sugar bisphosphate, $InsP_3$, $InsP_4$, $InsP_5$ and $InsP_6$ is 338.9888 (*m/z*, z=1), 418.9551 (*m/z*, z=1), 498.9214 (*m/z*, z=1), 288.9402 (*m/z*, z=2), 328.9234 (*m/z*, z=2), respectively. Theoretical mass to charge value for [$^{13}C_6$] Ins(1,2,3)P_3, [$^{13}C_6$] 2-OH $InsP_5$ and [$^{13}C_6$] $InsP_6$ is 424.9750 (*m/z*, z=1), 291.9503 (*m/z*, z=2), 331.9335 (*m/z*, z=2), respectively.

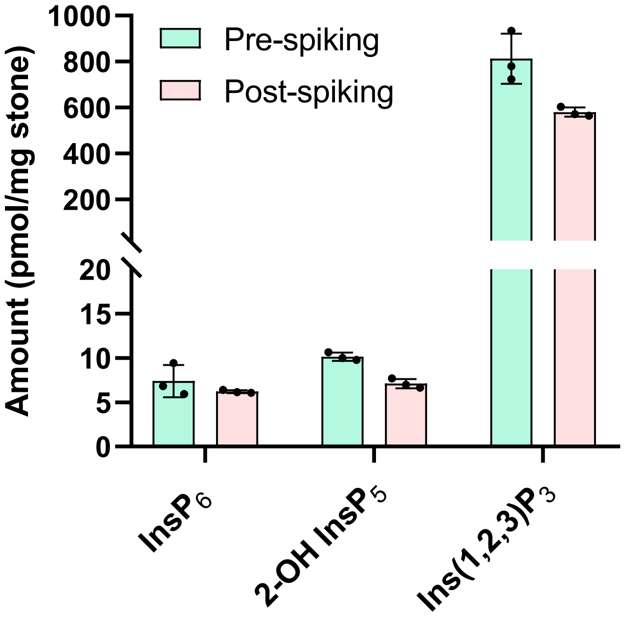

Figure S6 Recovery of $InsP_6$, 2-OH $InsP_5$ and Ins (1,2,3) P_3 from kidney stone 2 extracts with the TiO_2 extraction procedure. 2.5 μM [$^{13}C_6$] 2-OH $InsP_5$, 2.5 μM [$^{13}C_6$] $InsP_6$ and 19.33 μM [$^{13}C_6$] Ins (1,2,3) P_3 were added into kidney stone powder before the extraction (pre-spiking), or spiked into kidney stone extracts before measurement (post-spiking). The results showed good recovery for $InsP_6$ (84%), 2-OH $InsP_5$ (70%), and Ins (1,2,3) P_3 (71%). Data are means ± SD from three independent experiments.

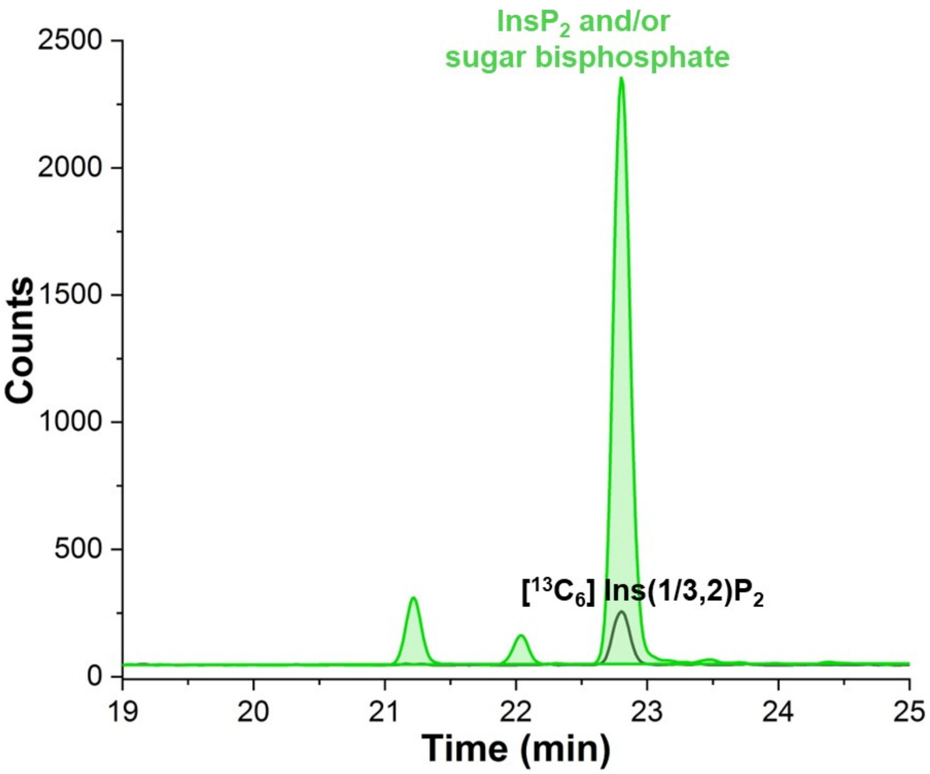

Figure S7 The most abundant peak (t_r ca 22.9 min) with *m/z* 338.9890 (z=1) has an identical migration time with [$^{13}C_6$] Ins (1/3,2) P_2 (black line) generated by pyrohydrolysis from [$^{13}C_6$] Ins (1,2,3) P_3 ,indicating Ins (1,2) P_2 and / or Ins (2,3) P_2 are present in kidney stones.

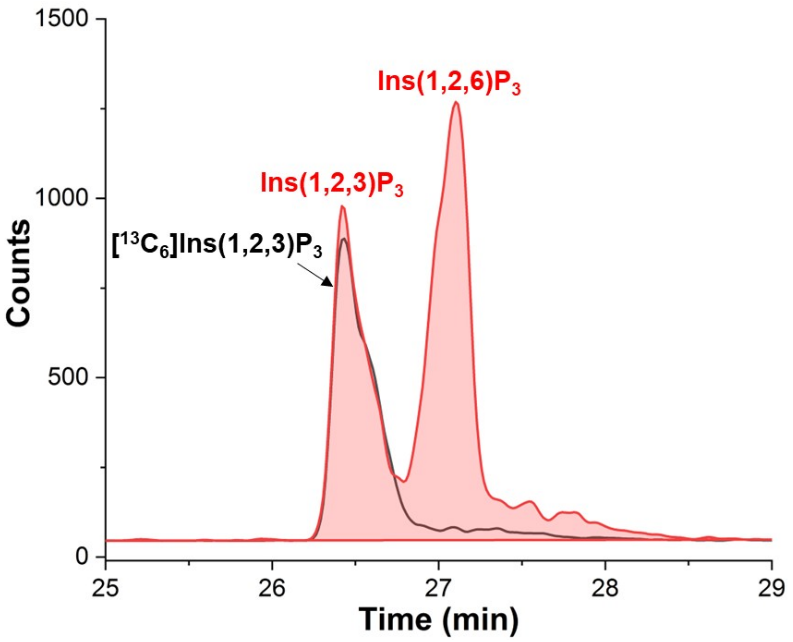

Figure S8 $InsP_3$ in kidney stone 1 spiked with Ins (1,2,6) P_3 standard. The $InsP_3$ isomer in kidney stone 1 migrated after Ins (1,2,3) P_3 and has the same migration time as the Ins (1,2,6) P_3 standard.

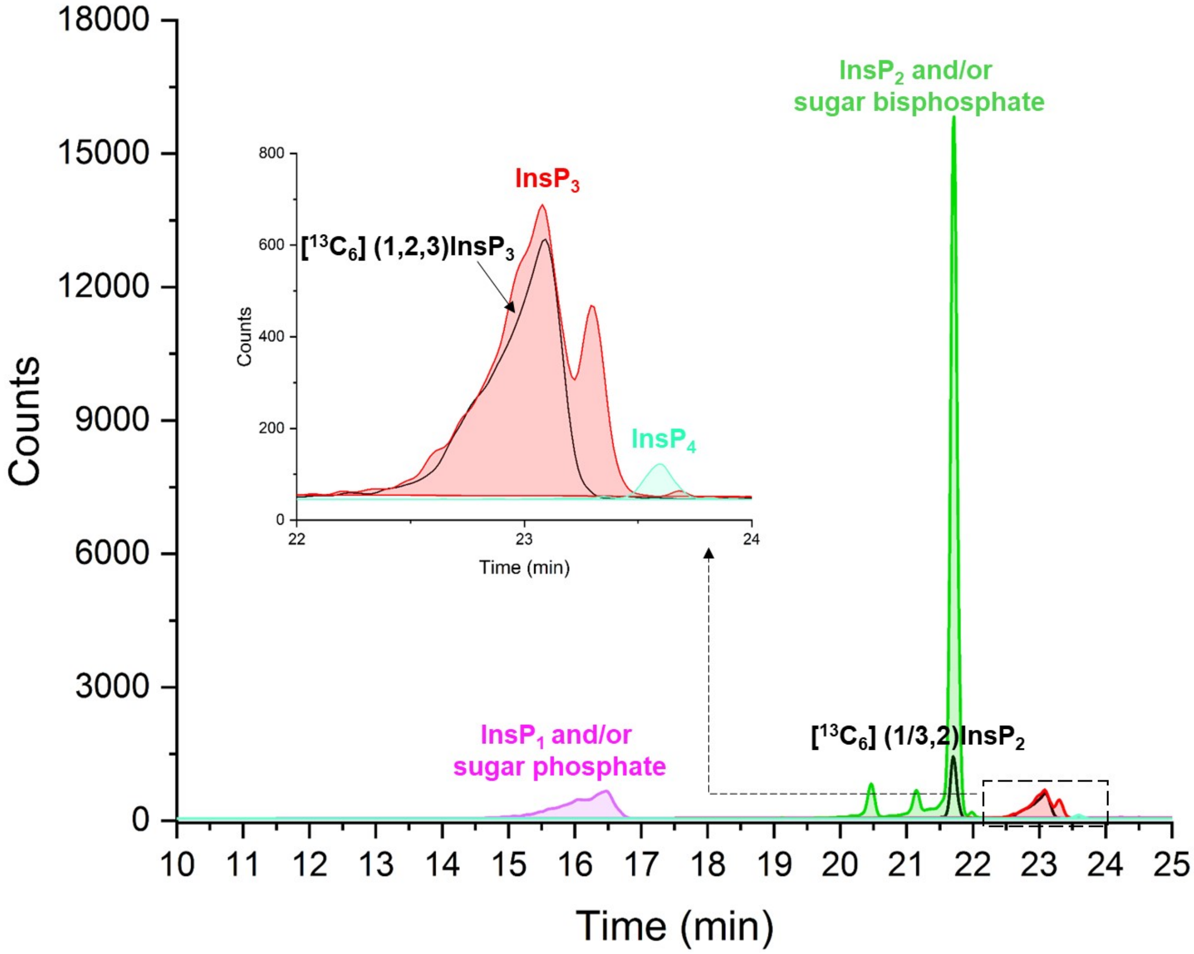

Figure S9 Extracted ion electropherograms of InsPs in urine from a healthy person by CE-QQQ. The InsPs were assigned by identical migration time with spiked $^{13}C_6$ labelled standards. Accurate masses of these InsPs and/or sugar phosphate were confirmed by CE-qTOF. [$^{13}C_6$] InsPs: black solid line, $InsP_4$: light blue trace, $InsP_3$: red trace, $InsP_2$ and /or sugar bisphosphate: green trace, $InsP_1$ and/or sugar phosphate: purple trace.

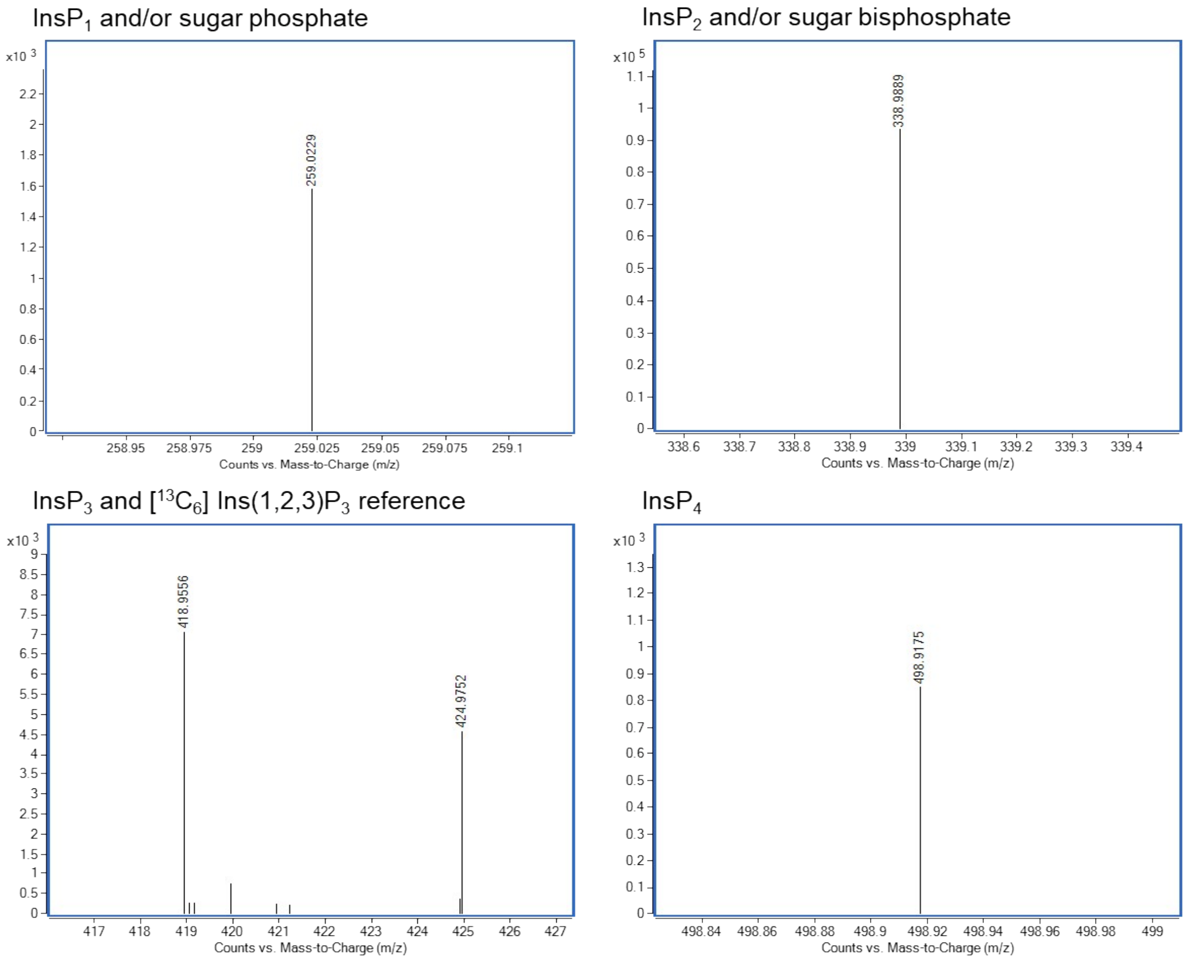

Figure S10 High-resolution mass spectra of InsPs from CE-qTOF analysis of urine from a healthy person spiked with [$^{13}C_6$] InsPs reference. Theoretical mass to charge value for $InsP_1$ or sugar phosphate, $InsP_2$ or sugar bisphosphate, $InsP_3$, $InsP_4$ is 259.0224 (*m/z*, z=1), 338.9888 (*m/z*, z=1), 418.9551 (*m/z*, z=1), 498.9214 (*m/z*, z=1), respectively. Theoretical mass to charge value for [$^{13}C_6$] Ins (1,2,3) P_3 is 424.9750 (*m/z*, z=1).

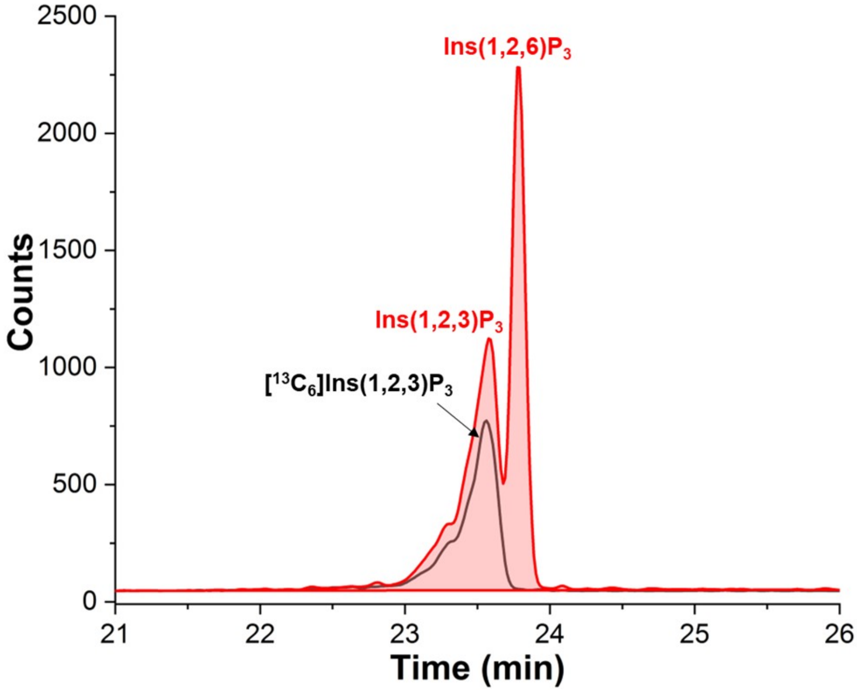

Figure S11 $InsP_3$ in urine from a patient spiked with Ins (1,2,6) P_3 standard. The $InsP_3$ isomer in kidney stone 1 migrated after Ins (1,2,3) P_3 and has the same migration time as the Ins (1,2,6) P_3 standard.

Reference:

[1] M.S. Wilson, S.J. Bulley, F. Pisani, R.F. Irvine, A. Saiardi, A novel method for the purification of inositol phosphates from biological samples reveals that no phytate is present in human plasma or urine, Open Biol 5(3) (2015) 150014. https://doi.org/10.1098/rsob.150014.
